# Neovascularization in Left Atrial Myxoma

**Published:** 2012-12-15

**Authors:** Laxman Dubey, Amit Kumar Chaurasia

**Affiliations:** 1 Department of Cardiology, College of Medical Sciences & Teaching Hospital, Bharatpur-10, Nepal

**Keywords:** Myxoma, Neovascularization, Coronary Angiography

## Abstract

**Abstract:**

We report a case with a left atrial mass who underwent coronary angiography to rule out coronary artery disease. Coronary angiography revealed an anomalous tortuous vascular structure originating from the left circumflex coronary artery to the left atrial tumor suggestive of neovascularization. Preoperative coronary angiography is useful for coronary artery evaluation and also provides additional information regarding the feeding vessel supplying the mass.

## 1. Introduction

Primary cardiac tumours are rare with an autopsy frequency of only 0.001-0.03% (1). Cardiac myxomas are the commonest form of primary cardiac tumors in adults and accounts for nearly half of all benign cardiac tumors (2). In a report of 323 consecutive patients undergoing surgical resection of primary cardiac tumours between 1957 and 2006 at the Mayo Clinic, 94% were benign and half of these were myxomas (3).

Myxomas are generally localized in the atria, mostly in the left atrium (LA) (2). Patients with myxomas are usually asymptomatic, but may have triad of embolism, intracardiac obstruction, and constitutional symptoms (4).

Tumour neovascularisation in cardiac myxomas has been shown in previous case reports. In our case report, we demonstrated a LA mass after transthoracic echocardiography, and during preoperative evaluation, coronary angiography (CAG) revealed the presence of tumour neovascularisation from the left circumflex coronary artery, with no evidence of coronary artery stenosis. Pathological analysis confirmed the clinical diagnosis of LA myxoma. To our knowledge, this is the first case of neovascularized LA myxoma reported from Nepal.

## 2. Case Report

**Figure 1:**
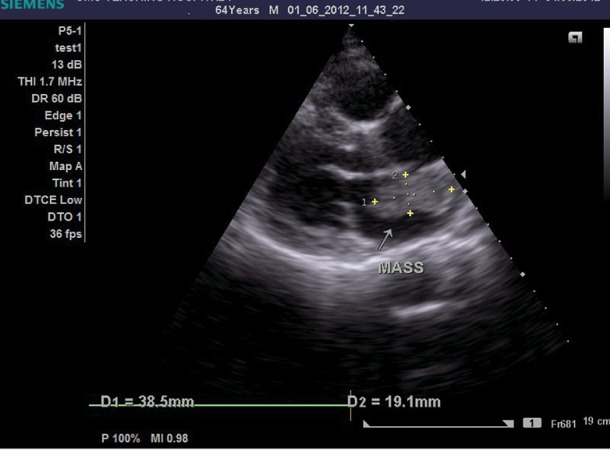
Transthoracic Echocardiography in Parasternal Long Axis View Showing a Heterogenous Mass in the Left Atrium Attached to the Interatrial Septum

**Figure 2:**
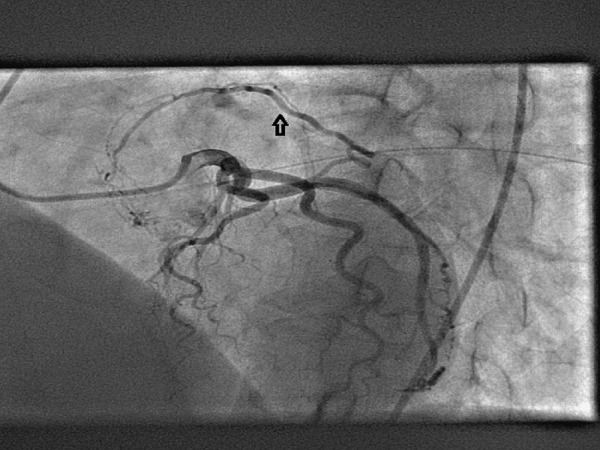
Selective Coronary Angiography of the Left Coronary System Showing a Feeding Artery (Arrow) Originating From the Left Circumflex Artery Supplying the Left Atrial Mass Visualized in the Early Phase

**Figure 3:**
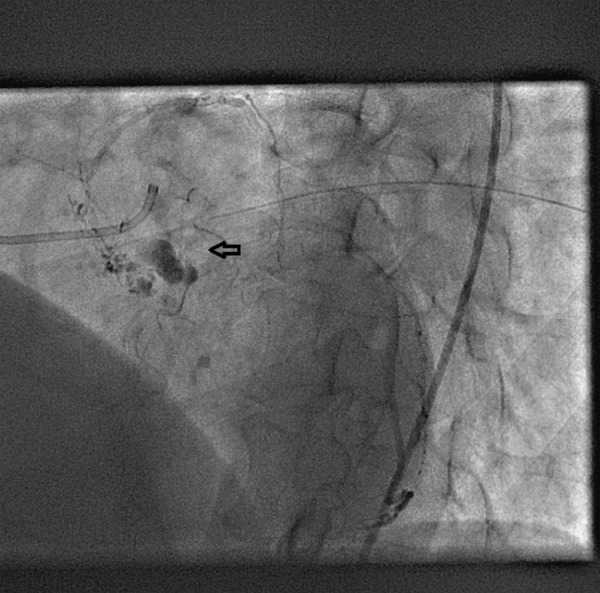
Left Atrial Mass (Arrow) Stained with Contrast Injected in the Left Coronary Artery System, Excellently Imaged Due to the Marked Contrast Enrichment in the Late Phase

A 64-year-old man was referred to our department for transthoracic echocardiography. He had a history of ischemic stroke 2 years back with left hemiparesis. The physical examination revealed a low-pitched sound heard during mid-diastole. Transthoracic echocardiography showed a large gelatinous mass measuring 38 x 19 mm in the LA arising from the interatrial septum (Figure 1). Coronary angiography was performed to rule out significant coronary artery stenotic lesions before surgery, which demonstrated the large feeding vessel arising from the proximal left circumflex artery (Figure 2) that supplied the mass (Figure 3) with no evidence of coronary artery stenosis. The mass was excised and the patient recovered uneventfully. Pathological analysis of the mass confirmed the clinical diagnosis of LA myxoma.

## 3. Discussion

Myxomas are the commonest primary tumours of the heart that have a typical echocardiographic appearance characterized by a gelatinous and friable mass (2). Myxomas are usually localized in the atria, majority in the LA. In rare cases, there is prominent vascularization of myxomas by supplying vessels from the coronary arteries. During CAG such neovascularization is usually verified, also three-dimensional imaging of the feeding vessels using multislice computed tomography has been reported (5). In our case, neovascularization of a LA myxoma from the left circumflex coronary artery was demonstrated in the CAG. This case demonstrated that CAG is important for ruling out coronary artery disease, and also can provide additional information to echocardiography for the diagnosis and evaluation of atrial myxoma by visualizing possible tumour neovascularization from coronary arteries, necessitating a surgical procedure. Presence of a large feeding artery substantially changes the operative strategy because large feeding arteries must be ligated in addition to excision of the myxoma.
